# The Keystone Veterinary Medical Association

**Published:** 1890-05

**Authors:** W. H. Ridge

**Affiliations:** Secretary


*The Keystone Veterinary Medical Association.*—This association held its monthly meeting at the College of Physicians, Philadelphia, April 5th, 1890. Dr. Huidekoper presided. The following members were present: Drs. Hoskins, Huidekoper, Keelor, Glass, Vandegrift, Williams, Cullen, Zuill, Webster, Eves, Ridge, Felton, Hartman, Lusson, Magill, Werntz and Birch. The minutes of the March meeting were read and approved. Dr. Goentner and Dr. Maurice, the essayists, were absent.

Dr. Werntz reported a case simulating rabies. A cow which was apparently all right early in the morning, during the forenoon gored the other cows and acted wildly, after which she was in a violent delirious condition. The next morning she died. Dr. Ridge cited similar cases, and thought it common among cows at this time of the year, and that it was due to impaction of the omasum. Dr. Vandegrift has had many similar cases; he also thought it due to impaction.

Dr. Glass related a case which he thought was rabies at the time. The stables were surrounded by a stone wall, and the cow had not been outside of it for several months. Dr. Glass advanced the idea that rabies is oftentimes due to a climatic influence, but was also communicable by inoculation; he gave an instance where a litter of pups had rabies, where there had been no chance of inoculation; one had it in the dumb form, another in the acute, and the third had it in the paralytic form. He was sure there was no communication with other dogs. In the fall of 1888 he had six cases of rabies in one month, one a little poodle that had not been out of the room for over a year, but had dumb rabies. He thinks that some miasmatic condition connected with the abnormal way in which the animal was housed produced the disease. *Apropos* of the value of the owner's word, Dr. Magill reported a case where a gentleman brought a dog to him saying it had swallowed the bath sponge. On examination he found the bitch was pregnant, which he told the owner, who said it could not be, as the bitch had never been out of the house and had never been with a dog. But in five weeks she had a litter of mongrel pups. He asked if this was due to climatic influence?

Dr. Glass reported five cases of docking with a new docking knife; the first two did well, the third died of tetanus in twenty-four hours, while the fourth and fifth had tetanus, but recovered. He said that he had always used another knife in operating before, and always with good results.

Dr. Lusson cited a case of keratitis where he used the massage treatment recommended by Dr. de Schweinitz, which consists of placing a small portion of yellow oxide of mercury in the eye and then rubbing the lid over the cornea for fifteen minutes daily. In two weeks he reduced the inflammation to a very small point, and thinks it very good treatment.

The secretary was instructed to write to Drs. Goentner and Maurice, the essayists, asking them to give their reasons for disappointing sixteen members by not being present when appointed to read papers.

W. H. RIDGE, *Secretary.*

